# P-164. Comparing long-term cardiovascular, neuropsychiatric, and autoimmune complications following dengue infection versus SARS-CoV-2 infection

**DOI:** 10.1093/ofid/ofae631.369

**Published:** 2025-01-29

**Authors:** Jue Tao Lim

**Affiliations:** LKCMedicine, NTU, Singapore, Singapore

## Abstract

**Background:**

While persistence of chronic symptoms following dengue infection has been documented in small prospective cohorts, population-based studies are limited. The post-acute risk of new-incident multi-systemic complications following dengue infection was evaluated and contrasted against that following SARS-CoV-2 infection in a multi-ethnic adult Asian population

**Methods:**

National testing and healthcare claims databases in Singapore were utilised to build a retrospective population-based adult cohort with laboratory-confirmed infection during overlapping waves of SARS-CoV-2 and dengue transmission (1 Jul 2021-31 Oct 2022). Risks of new-incident cardiovascular/neuropsychiatric/autoimmune complications 31-300 days post-dengue infection, contrasted with SARS-CoV-2 infection were estimated using Cox regression with overlap weights. Risks were reported in terms of adjusted-hazard-ratio (aHR) and excess-burden (EB) per-1000-persons.

**Results:**

11,960 dengue-infected individuals and 1,265,329 contemporaneous COVID-19 cases were included; the majority had mild initial infection not requiring hospitalisation. Amongst dengue-infected individuals, there was 50% (aHR=1.50 [1.25–1.81]) increased risk of any sequelae (EB=1.51[1.40-4.42]), with 90% (aHR=1.90 [1.44–2.52]) increased risk of cardiovascular sequelae (EB=1.64[0.09-3.37]). Specifically, increased risk of ischemic-heart-disease (aHR=1.88[1.31-2.70]), other cardiac disorders (aHR=2.87[1.74-4.74]) and thrombotic disorders (aHR=4.56[2.37-8.75]) was noted. Elevated risk of individual neurological sequelae, including cerebrovascular disorders (aHR=2.05[1.29-3.25]) and cognition/memory disorders (aHR=2.31[1.40-3.84]) was observed in dengue-infected individuals compared to COVID-19 cases. Elevated risks of post-acute sequelae in dengue survivors were observed when contrasted against COVID-19 survivors infected during Delta/Omicron predominance, as well as across vaccination strata.

**Conclusion:**

Increased risk of post-acute cardiovascular/neurological complications was observed in dengue survivors, when contrasted against COVID-19 survivors infected during Delta/Omicron predominance.

**Disclosures:**

**All Authors**: No reported disclosures

